# Optimized Ultrafiltration Membrane Based on Acrylic Fiber Waste for Organic Compounds Removal from Wastewater

**DOI:** 10.3390/membranes15120359

**Published:** 2025-11-28

**Authors:** Ahmed A. Bhran, Eman S. Mansor, Heba Abdallah, Abdelrahman G. Gadallah

**Affiliations:** 1 Chemical Engineering Department, College of Engineering, Imam Mohammad Ibn Saud Islamic University (IMSIU), Riyadh 11432, Saudi Arabia; agadallah@imamu.edu.sa; 2 Water Pollution Research Department, Environment and Climate Change Research Institute, National Research Centre, Giza 12622, Egypt; eman_mansor31@yahoo.com; 3 Chemical Engineering & Pilot Plant Department, Engineering Research Institute, New and Renewable Energy, National Research Centre, Giza 12622, Egypt; heba_nasr94@yahoo.com

**Keywords:** ultrafiltration, acrylic fiber waste, wastewater, organic removal

## Abstract

This study reports the development of an optimized tight ultrafiltration (UF) membrane prepared from recycled acrylic fiber (polyacrylonitrile, PAN) waste for the efficient removal of organic pollutants from water. Membranes were fabricated using different concentrations of acrylic fiber waste to examine the influence of polymer content on their morphology and performance. The prepared membranes were characterized using scanning electron microscopy (SEM), porosity measurements, contact angle analysis, and mechanical strength testing to evaluate their structural and physicochemical properties. Among the tested formulations, membrane M4, containing 22.5 wt.% acrylic fiber waste, shows the most balanced performance, high mechanical integrity, and good surface hydrophilicity, with a contact angle of about 52° and porosity of 27%. The optimized M4 membrane demonstrates excellent pure water flux of 65 LMH. M4 achieves a flux recovery ratio (FRR) above 80%. Its performance was further evaluated for the removal of humic acid (HA) and paracetamol as a model of organic contaminants. The results also demonstrate strong chemical stability under acidic and basic conditions, highlighting the potential of recycled acrylic fiber waste as a sustainable polymer source for high-performance tight UF membranes. This approach offers an environmentally friendly and cost-effective solution for water purification and pharmaceutical contaminant removal.

## 1. Introduction

Water pollution is a significant global issue, as the discharge of various pollutants into aquatic ecosystems poses serious health risks to humans. Based on the World Health Organization, the percentage of wastewater released into the environment without purification is about 44%, so around 2 billion people are dealing with polluted water, and more than 350 million people suffer health problems due to unsafe water usage [1,2]. Humic acid, a natural by-product of decomposed plant and animal material, is a common organic contaminant in surface and groundwater. It imparts color, odor, and taste to water and reacts with chlorine during disinfection to form harmful by-products such as trihalomethanes. In addition, humic acid promotes membrane fouling, reducing the efficiency of filtration systems. Excessive chlorine use in surface-water disinfection is problematic because residual chlorine reacts with humic acid to form disinfection by-products (DBPs). Prisciandaro and Mazziotti di Celso evaluated the feasibility of combining ultrafiltration (UF) with chlorination in a tangential flow mode [3]. Therefore, its effective removal is vital for producing safe drinking water and maintaining the performance of water treatment processes. One of the primary contributors to this problem is industrial wastewater, particularly due to the rapid advancement of industrial technologies. Among these, the pharmaceutical industries are especially problematic. Also, domestic sewage and hospital sewage contain amounts of pharmaceutically active compounds (PhACs) that adversely affect aquatic life, animals, plants, and human health. These contaminants, such as nonsteroidal anti-inflammatory drugs (NSAIDs), antibiotics, and especially paracetamol, a drug that is widely used. The continuous usage of paracetamol leads to persistence in water bodies and can tolerate conventional treatment even at low concentrations [4,5,6]. Various advanced methods have been developed to enhance the removal of the selected paracetamol as micropollutants from water, including adsorption, fenton, photocatalysis, and membrane filtration [7,8,9,10,11,12]. Membrane filtration removes contaminants of different sizes, from 1000 nanometers to less than 1nm. Using membranes with diverse polymeric materials has been applied for water purification and broad separation operations.

These polymers include polystyrene, polyethersulfone, polyacrylonitrile, polyvinyl diflouride, and cellulose acetate [13,14,15,16,17,18]. Among these polymeric materials, Polyacrylonitrile (PAN) stands out as one of the most utilized materials for water and wastewater treatment due to its excellent properties. PAN offers good chemical resistance, inherent hydrophilicity, and high solubility in organic solvents. Polyacrylonitrile ultrafiltration membranes have been enhanced using 3D-MXene for the removal of oil and dyes from wastewater [19]. Also, PAN Ultrafiltration Membrane was loaded with TiO_2_ for BSA removal and to enhance antifouling performance [20]. Additionally, acrylic fibers as polymer waste present an alternative source for membrane materials. Acrylic fibers are relatively low-cost copolymers, primarily consisting of around 85% acrylonitrile, along with other monomers like methyl acrylate. Due to their excellent mechanical properties, waste acrylic fibers have been utilized to enhance the strength of granular soils [21,22]. In addition, membrane-based PAN often suffers from fouling, which reduces permeability and increases operational costs. We believed that using acrylic fiber waste as a polymeric matrix would not only enhance the hydrophilic properties but also strengthen the mechanical characteristics of these membranes.

This study introduces a promising membrane fabricated by incorporating 22.5 wt.% of acrylic fiber waste, using dimethyl formamide and water as solvents, via non-solvent induced phase separation (NIPS). This work exploits the membrane for the selective removal of paracetamol as PhACs. The resulting membranes exhibit superior hydrophilicity, porosity, mechanical, and antifouling properties, enabling efficient removal of organic pollutants. This study bridges the gap between material innovation and practical implementation. The findings offer a sustainable and high-performance solution for wastewater treatment and organic pollutant remediation.

## 2. Materials and Methods

### 2.1. Materials

Acrylic fiber waste (ACF) was collected from a local textile factory. N, N-dimethylformamide (reagent grade, 99%) was purchased from Merck & Co., Inc. (Rahway, NJ, USA). Humic acid (HA) powder was delivered from Loba Chemie Pvt. Ltd. (Mumbai, India) and was used as an organic pollutant to evaluate the fouling behavior and filtration efficacy. Glacial hydrochloric acid (ACS reagent, 37%) and sodium hydroxide with concentration ≥98% were supplied from Loba Chemie Pvt. Ltd. (Mumbai, India) and were used to adjust the pH of the solution. Paracetamol (Acetaminophen, N-acetyl-p-aminophenol) was purchased from Sigma-Aldrich Inc. (St. Louis, MO, USA).

### 2.2. Preparation of Acrylic Fiber Membranes

**Figure 1:**
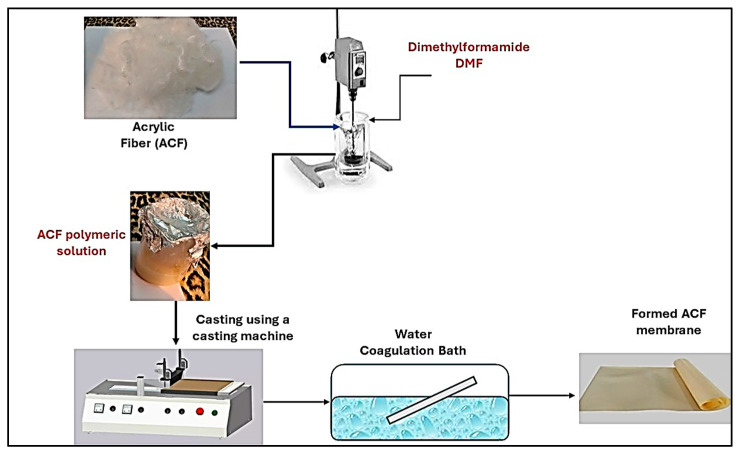
ACF membrane preparation steps.

UF Acrylic Fiber membranes with different weight percentages were prepared as shown in Table 1. The polymeric solution was stirred until dissolved and then was cast on a clean, dried glass plate by using a film applicator at 250 μm constant thickness. After that, the phase inversion process was carried out by immersion of the glass sheets in a water bath. Figure 1 indicates the ACF membrane preparation steps.

### 2.3. Membrane Characterization

A scanning electron microscope was utilized to assess the morphological features of the membrane samples. Before analysis, the membrane-based samples were sputter-coated with gold to avoid charging. SEM images were captured by a Quanta FEG-250 scanning electron microscope (FEI Company, Hillsboro, OR, USA) at an accelerating voltage of 20 KV. The contact angle measurements were recorded for all membranes using SCA 20, OCA 15 EC, Data Physics Instrument (Data-Physics Instruments GmbH, Filderstadt, Germany), to detect the hydrophilic characteristic of the surface of the membranes. A drop of water with a certain volume was dripped from the capillary needle of the device onto the sample surface. The image of the water drop on the membrane surface was then captured using a camera. According to the value of the contact angle (θ), the membrane textures can indicate the membrane surface nature, where θ > 90° represents a hydrophobic property, while represents θ < 90° a hydrophilic one. The test was replicated five times at different positions on the surface of the membranes, and the results were averaged for each sample. The mechanical properties of membranes in terms of ultimate tensile stress and strain at rupture were measured using an H5KS universal tensile testing machine (Tinius Olsen Ltd., Salfords, UK). The samples of dimension 10 cm × 2.5 cm were tested, and five measurements were carried out. The average values were calculated for each sample. The viscosity measurement for the different weights of the acrylic fiber used was tested using a BROOKFIELD AMETEK DV2T viscometer (AMETEK Brookfield, Middleboro, MA, USA). The experiments were carried out using spindle number 27. The temperature was kept at 25 °C to prepare spinning solutions until every polymer was tested. The porosity for the fabricated membrane was determined using the dry-wet method. The samples were soaked in distilled water for 12 h, after that the wetted samples dried in an oven at 80 °C for 12 h. The porosity (ε) was calculated by Equation (1).

(1) $$\varepsilon \;(\%)=(\frac{W_{wet}-W_{dry}}{d_{w}Ah})\times 100$$

where ε is the membrane porosity, the wet weight is (*W_wet_*), the dry weight is (*W_dry_*) were weighted (g), *d*_w_ is the density of pure water (0.9980 g/cm^3^), A refers to the membrane area (cm^2^), and h refers to the membrane thickness (cm).

### 2.4. Organic Pollutants Removal and Membrane Filtration

Permeation tests of acrylic fiber membranes were conducted by filtering pure water flux using a dead-end filtration unit with a membrane area of 12.7 cm^2^. It was mounted in a module for testing. Flux-Q was quantified at a constant pressure of 5 bars. The permeated water through the membrane was collected, and the pure water flux was calculated using Equation (2).

(2) $$Flux=\frac{Q}{AT}$$

where *Q*, *A*, and *T* are the quantity of permeate (L), membrane area (m^2^), and sampling time (h), respectively.

The contaminated water was prepared by dissolving 50 mg/L of HA as an organic pollutant in the water. First, the filtration test was performed for the pure water test for 60 min at the applied pressure to reduce the impact of the membrane compactness, then the filtration test was performed using the HA solution. The experiment with the HA solution was carried out for 300 min. The permeated water through the membrane was collected at time intervals of 15 min. The permeation flux of the filtered water was measured according to Equation (2). Finally, the tested membranes were cleaned with distilled water as hydraulic cleaning for 60 min, and the effect of the cake layer on the washed membranes was detected by recording pure water flux for 30 min for each membrane. The main fouling parameters are the reversible fouling ratio (DR_r_), the flux recovery ratio (FRR), and the irreversible fouling ratio (DR_ir_), and they were calculated using Equations (3)–(5), respectively. The durability and fouling performance of the prepared membranes were evaluated by applying 500 mg/L of HA at 5 bars for 6 h. The filtration was applied for 6 h, and the water flux was calculated (Jp, LMH) [18]. Then, the tested membrane was hydraulically cleaned, and the water flux (J_WC_) was determined again as shown in the following equations:

(3) $${DR}_{r}(\%)=\left(\frac{J_{wc}-J_{p}}{J_{wI}}\right)\times 100$$

(4) $${DR}_{ir}(\%)=\left(\frac{J_{wI}-J_{Wc}}{J_{wI}}\right)\times 100$$

(5) $$FRR(\%)=\left(\frac{J_{wc}}{J_{wI}}\right)\times 100$$

where J_WI_ is the pure water flux of the clean (virgin) membrane obtained at the start of the fouling experiment. The stability of the prepared membranes towards acid and base solutions was studied through chemical cleaning. Both 0.1 N HCl and 0.1 N NaOH reagents were applied after filtration of the HA solution using the washed membranes. These reagents were used in pure water, and the pure water flux was recorded.

The performance of the tested prepared membranes for the first time, after hydraulic washing and after chemical washing towards the HA solution, was determined using Equation (6). Also, the optimized membrane was tested using 20 mg/L paracetamol as a model of PhAC. The removal efficiency was obtained using Equation (6):

(6) $$Removal\left(\%\right)=\left(1-\left(\frac{C_{P}}{C_{F}}\right)\right)\times 100$$

where C_P_ is the HA and APAP concentration (mgL^−1^) in the produced solution after filtration, and C_F_ is the concentration of these pollutants in the starting solution. The tested pollutant concentration was analyzed using a UV–vis spectrophotometer (Agilent-Cary 100, Agilent Technologies, Santa Clara, CA, USA). It should be noted that the standard deviations were calculated based on triplicate measurements, with an allowable range of ±3–5% for retention rates.

## 3. Results and Discussions

### 3.1. Characterizations of UF Acrylic Fiber Membranes

#### 3.1.1. The Viscosity of the Different Weights of ACF

To study the impact of viscosity of ACF polymeric solutions, the ACF weight concentration range of 15–22.5 g was used. Table 2 shows that with an increase in the weight percent of the polymer from 15 to 22.5 wt.%, the viscosity of the solution increases from 278 to 4030 CP, respectively. This increase is more than 14 times. So, low concentrations of ACF as the main polymer are not suitable for forming a tight UF membrane. From Table 2, the trend of the increasing viscosity was clear, due to the increment with polymer chain entanglement that causes an increase in the viscosity [23,24].

In this study, all prepared membranes exhibited the characteristic asymmetric structure typical of phase inversion processes. The internal morphology—ranging from spongy to finger-like formations—was strongly influenced by both the polymer concentration and the viscosity of the casting solution. As the polymer content increased, the formation of macrovoids decreased, resulting in smaller pores. Consequently, the membrane prepared with 22.5% acrylic showed the lowest porosity among the tested samples. The polymeric solution promoted an effective mass transfer between the solvent within the cast polymer film and the nonsolvent (water) in the coagulation bath during membrane formation, which provides a less porous structure, small pores, and fewer.

#### 3.1.2. The Surface and Cross-Section Morphologies of Membranes

**Figure 2:**
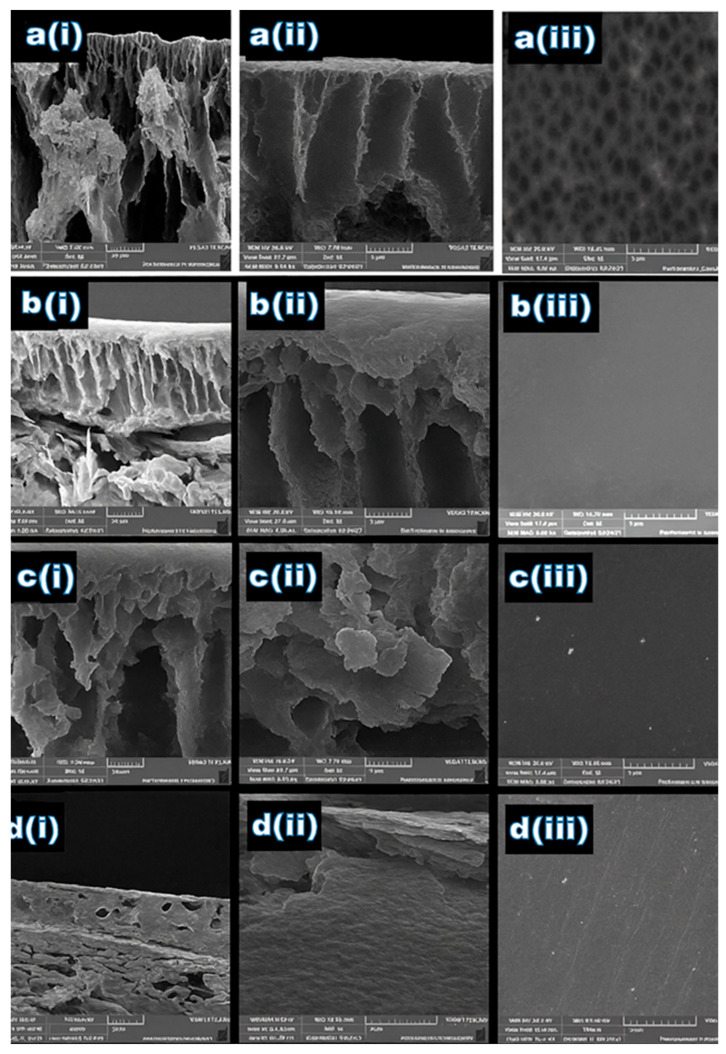
The SEM images of ACF membranes at different weight concentrations: (**a**) 15 wt.%, (**b**) 17.5 wt.%, (**c**) 20 wt.%, and (**d**) 22.5 wt.% for (**i**) cross-section at 20 µm magnification, (**ii**) cross-section at 5 µm magnification, and (**iii**) surface layer with 5 µm magnification.

Figure 2 Present the cross-sectional and surface morphology images of the prepared acrylic fiber membranes. The impact of different weight percentages of ACF polymer waste on the textural properties of the prepared membrane can be detected from the presence of small or big macrovoids in the substructure, the formation dense layer. Hypothetically, the dense layer can be formed by raising the weight percent of polymer, as well as the structure of the macrovoids– fewer and smaller voids [25]. It is clear that all the fabricated ACF membranes have an asymmetric structure with a finger-like structure and that the macrovoids vary relative to the weight percent of ACF polymer waste. The finger-like sub-layer is found in prepared membranes with a lower weight percent (15–17.5 wt.%). Presence of the largest finger-like macrovoids in the sublayer with many pores is attributed to the rate of diffusion for the distilled water into the phase of poor polymer which is faster than the rate of DMF diffusion outward [26]. So, this porous sublayer structure is owed to the lower weight percent of ACF. On the other hand, for membranes prepared with a weight percent of 20 and 22.5 wt.%, (Figure 2(c(ii),d(ii))), a dense layer was also observed. The formation of the thin skin layer is due to the instantaneous demixing of DMF and distilled water during the solidification process of the dope solution and the hydrophilic nature of ACF. As shown in Figure 2(d(ii)), the M4 membrane containing 22.5 wt.% acrylic exhibited the most compact dense layer, characterized by the smallest voids and the lowest surface porosity [27].

#### 3.1.3. Mechanical Properties

**Figure 3:**
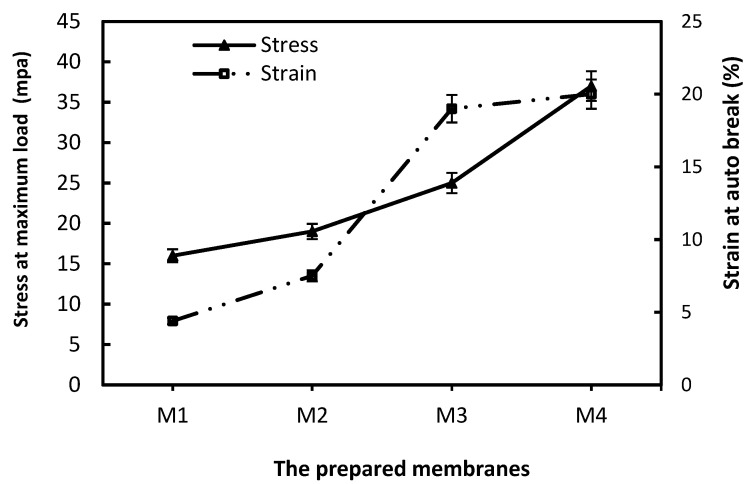
Mechanical properties of asymmetric ACF Membrane at various weight percentages.

The fabricated ACF membranes with different polymer concentrations had good mechanical properties in terms of stress and strain. Figure 3 presents the results of the mechanical properties for the fabricated membranes based on ACF (M1–M4). From Figure 3, M1 had low stress properties owing to the lowest polymeric weight percentage and lower polymer-chain interactions. So, from the obtained data, an increase in the weight percent of ACF leads to the enhancement of the stress of the membranes. As shown in Figure 3, the stress of the membrane is enhanced by about two times as the weight percent is raised from 15 to 22.5 wt.%. This enhancement in stress is owed to the improvement in the substructure of the membrane, the best interaction, and the uniformity of polymer chains. This result is confirmed by SEM images of the fabricated membranes; the presence of macrovoids becomes smaller as the weight percent of ACF approaches 22.5. So, raising the weight percent of polymer affected the stress due to the formation of fewer macrovoids [24,28]. However, the strain results for the membranes showed a slightly different behavior towards the weight percent of ACF. By raising the weight percent of ACF from 15 to 20 wt.%, the strain of the membranes was enhanced. Then, with a weight percent of 22.5, the strain reduced slightly. The strain properties represent the ability of the membrane to be stretched before the point of break. This behavior was recorded with Ismail et al. [29] when using polysulfone with a weight percent 30%, a decline in elongation was observed. From the SEM analysis and mechanical test, we demonstrated the optimum membrane with a polymeric weight of 22.5 wt.% for the acrylic membrane [29].

#### 3.1.4. Porosity and Contact Angle Results

Table 3 presents the porosity results and water contact angle measurements for the fabricated membranes, varying the weight load percent of ACF. The contact angle of M1 was 41.8 degrees, which is the lowest contact angle compared to the others. But the difference in contact angle results is not significant with the highest weight percent of AFC having 52.1 degrees. So, the results demonstrated a hydrophilic surface where all the membranes have a contact angle less than 60°. On the other hand, the porosity decreased from M1 to M4 54.1% to 27%. This decline highlights the enhanced surface skin layer achieved by 22.5 wt.% for the polymer matrix. This was due to raising the weight of the polymer, which reduced the internal size of the pores and the distance between spherulites. So, good nucleation density increases as spherulite numbers increase with 22.5 wt.% polymer concentration, and therefore porosity decreases. This trend was observed in the work of Akbari and Yegan [30]. The SEM images also confirmed the presence of a porous top surface for M1 with 15 wt.%, as shown in Figure 2(a(iii)). Considering the SEM, mechanical, porosity, and contact angle results, the optimum membrane is M4 with 22.5 wt.%.

### 3.2. Performance of the Membrane Towards Organic Compounds Removal

**Figure 4:**
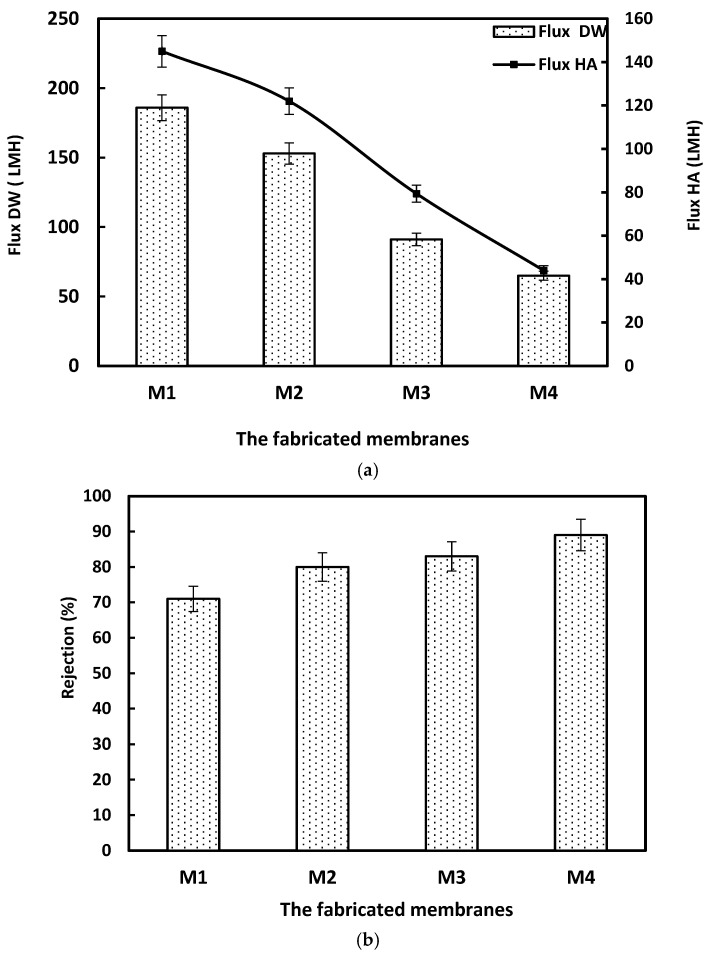
(**a**). The data of the permeation flux test for the fabricated membranes. (**b**). The rejection of the fabricated membranes towards the HA solution. Error bars represent the standard deviation from triplicate measurements, with an allowable range of ±3–5% for retention rates.

Figure 4a illustrates the permeation flux of the fabricated membranes containing different weights of ACF polymer waste. The flux for distilled water and humic acid solution (50 ppm) was measured using a filtration system for 60 min and 90 min for DW and HA, respectively. The permeated water was collected every 15 min to calculate the flux in LMH. The permeation fluxes of the membranes when performed for pure water and HA were decreased as the weight percent of ACF was increased. The pure water flux of the M1 membrane decreased from 186 to 65 LMH in M4. The same reduction was obtained in permeate flux during filtering the HA solution, reaching 145 and 44 LMH for M1 and M4 membranes, respectively. This reduction trend was accompanied by an improvement in the rejection percent of the HA solution. The rejection increased from 71 to 89% as the weight of polymer was raised from 15 to 22.5 wt.%, as shown in Figure 4b [31].

**Figure 5:**
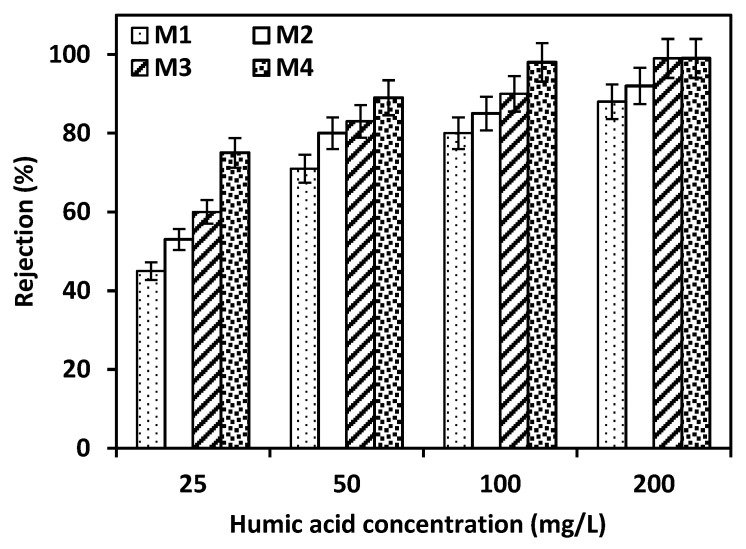
The effect of different concentrations of HA solution on the rejection of the fabricated membranes. Error bars represent the standard deviation from triplicate measurements, with an allowable range of ±3–5% for retention rates.

The formation of a skin-dense layer on the surface of M4 promoted the membrane to have the highest rejection for HA. To confirm the best efficiency for M4, the fabricated membranes were evaluated using different concentrations of humic acid from 25 to 200 mg/L, as presented in Figure 5. As the concentration of the HA solution increased, the rejection percent for all membranes (M1–M4) was improved. This trend is typical for membranes with charge-repulsion or size-exclusion mechanisms, where higher solute concentrations increase the apparent rejection due to membrane saturation or surface interaction enhancement [32,33]. However, with the lowest concentration of HA solution (25 ppm), the membrane with a weight percent of 22.5 (M4) is still highlighting a significant impact of the dense layer on membrane selectivity. M1 has the lowest percent removal for HA solution (25 ppm), reaching 45% and this percent increased with M4, reaching 79%. It confirms that the porosity for M4 declined as presented in Table 3, and the skin layer became more compact along with the increase in ACF concentration as presented in the SEM images.

**Figure 6:**
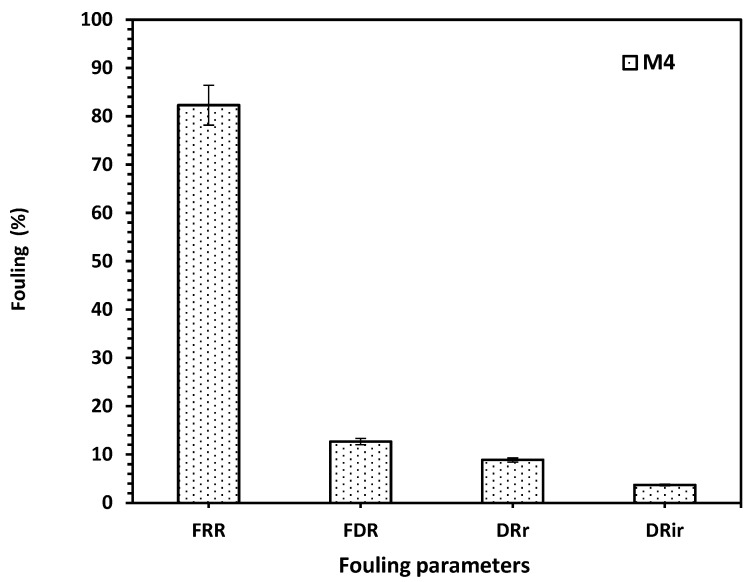
The fouling performance analysis of the M4 membrane, expressed as percentages for FRR, FDR, DR_r_, and DR_ir._

The antifouling performance of the optimized membrane based on acrylic fiber is presented in Figure 6. M4 has an excellent FFR ratio of about 83% where FFR represents how well the membrane recovers its permeability after cleaning. Also, the Flux Decline Rate (FDR), 12.7% confirms that fouling develops slowly, so M4 has a stable flux over time. In addition, the values of DRr and DR_ir_ are lower, indicating that foulants can be easily removed and the M4 membrane has strong chemical and structural stability, with little irreversible pore blocking or adsorption. Thus, M4 is a promising membrane for wastewater treatment or reuse applications, especially in systems prone to organic or colloidal fouling.

**Figure 7:**
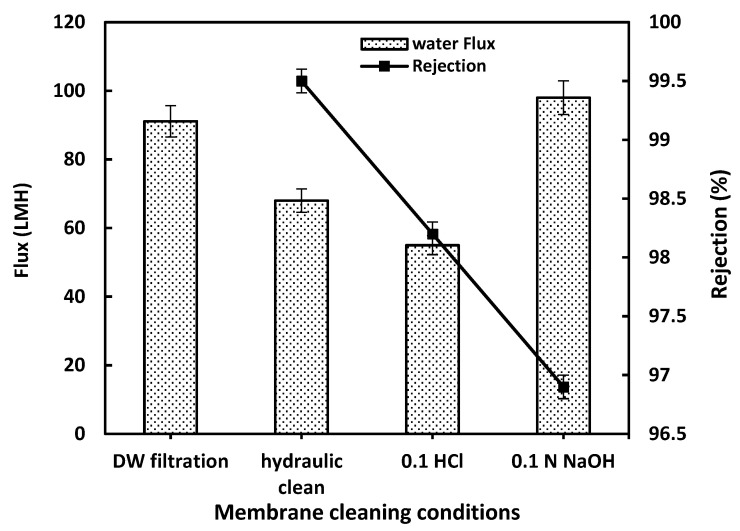
The effect of acid-based cleaning on the M4 membrane performance.

During HA filtration, rejected organic compounds deposit on the membrane surface and within its pores. So, the tested optimized membrane was evaluated towards acid cleaning with HCl and Base cleaning with NaOH, targeting organic fouling. Also, this performance using acid-based cleaning helps determine its chemical stability and reusability. Acrylic fiber (PAN)-based membranes are known for their good chemical resistance, but they can be sensitive to alkalis. As shown in Figure 7, the optimized M4 membrane is stable under acidic cleaning and hydraulic cleaning. Under basic conditions, a significant flux recovery was obtained owing to the partial hydrolysis of nitrile groups (–C≡N) that may occur, forming amide (–CONH_2_) or carboxylate (–COO–) groups. This behavior had an effect on the contact angle value and led to an enhancement of the hydrophilicity due to surface activation (formation of amide/carboxyl groups). So, the acid-based cleaning process effectively saves the M4 rejection performance for the HA solution without significant deterioration, reaching more than 96.5% as shown in Figure 7. This makes acrylic fiber waste-based membranes a sustainable and robust material for wastewater treatment, combining high flux recovery, antifouling performance, and chemical resilience.

**Figure 8:**
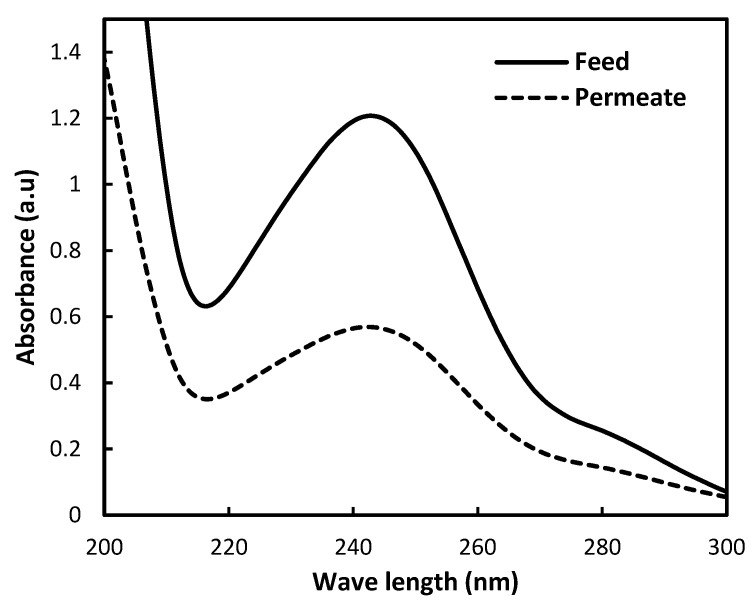
UV–Vis spectrum for the removal of paracetamol using M4.

To ensure the efficiency of the prepared M4, the membrane was tested using paracetamol. The strong peak around 245 nm is typical for the benzene ring conjugated with a hydroxyl and amide group. As shown in Figure 8, the absorbance intensity of the permeate is significantly lower than that of the feed. This indicates that the M4 membrane has good efficiency towards paracetamol. The spectral shape shows that the removal of paracetamol occurs by physical rejection or adsorption, with no degradation during filtration. This result supports the potential of waste-based membranes for micropollutant removal from wastewater, particularly pharmaceutical residues, where the removal percentage of paracetamol reached 54%.

A comparison of the fouling parameters of the optimum M4 ACF membrane with those of previously reported PAN-based and commercial membranes highlights its strong antifouling behavior. The ACF (22.5%) membrane developed in this work achieves an FRR of 82.3%, together with low reversible (8.9%) and irreversible (3.7%) fouling. These results indicate that the addition of ACF provides a favorable balance of fouling resistance and membrane stability as presented in Table 4. In contrast PAN membranes P (AN-co-MA) developed for oil/toluene separation showed less values of IFR, and FRR ~21%, and 79%.; commercial membranes such as PES, PVDF, and PS often exhibit much poorer resistance to fouling, with FRR values reported at only 11.1%, 8.6%, 7.9%, and 6.2% for PS, PVDF, PES, and PAN, respectively, when tested in digestate ultrafiltration [34,35,36,37].

## 4. Conclusions

In this study, tight ultrafiltration (UF) membranes were successfully fabricated from recycled acrylic fiber (polyacrylonitrile) waste, demonstrating the potential of converting industrial textile residues into high-performance filtration materials. Systematic variation in the polymer concentration showed that the membrane containing 22.5 wt.% acrylic fiber waste (M4) offered the optimal balance between structural and functional properties. The polymeric matrix for this percentage reached the highest viscosity value of 4030 CP at 50 rpm. The M4 membrane exhibited an interconnected, less porous morphology, strong mechanical stability reaching 24 MPa for stress and 17% for elongation, and an enhanced hydrophilicity to reach 52 degrees for M4, which together resulted in high pure water flux and excellent antifouling performance. The flux recovery ratio (FRR) is above 80%, along with FDR, DRr, and DRir values of 12.7%, 8.9%, and 3.7%, respectively. The application of two models of organic pollutants demonstrated outstandingly high efficiency in removing humic acid and good removal for paracetamol from aqueous solutions, confirming its effectiveness in treating water contaminated with organic micropollutants. Overall, the results highlight the potential of acrylic fiber waste as a valuable raw material for producing advanced separation membranes, promoting both environmental sustainability and resource recovery. The developed membrane represents a promising, low-cost, and eco-friendly solution for water purification and wastewater reuse applications.

## Figures and Tables

**Table 1:** Composition of the fabricated acrylic fiber membranes.

Membranes	ACF (wt.%)	DMF (wt.%)
M1	15	85
M2	17.5	82.5
M3	20	80
M4	22.5	77.5

**Table 2:** The viscosity and other physicochemical properties of the acrylic polymer solution.

Membrane	Viscosity (cp)	Shear Stress (dyn/cm^2^)	Shear Rate (1/s)	Torque (%)	Speed (rpm)
M1	278	190	68	22.3	200
	285	146	51	17.3	150
	292	99	34	11.7	100
M2	585	398	46.5	50	200
	560	290	51	34	150
	560	191	34	23	100
M3	-, *	-	-	-	200
	1528	780	51	92	150
	1530	519	34	61	100
M4	-	-	-	-	200
	-	-	-	-	150
	4030	685	17	63	50

* The “-” entries mean that for membranes at 150 and 200 rpm, the experimental measurements (viscosity, shear stress, shear rate, and torque) were not recorded (out of range) due to instrument limits at this speed.

**Table 3:** Water contact angle and porosity for prepared membranes.

Membrane	Contact Angle (Degree)	Porosity (%)
M1	41.8° 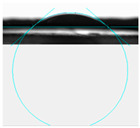	54.1%
M2	48.4° 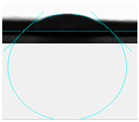	46.6%
M3	50.5° 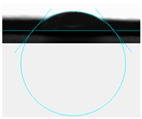	34.2%
M4	52.1° 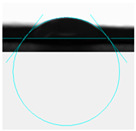	27.0%

**Table 4:** Comparison of fouling parameters of the prepared membrane with commercial membranes.

Membrane Description	Fouling Parameters	**Ref.**
Polyacrylonitrile (PAN) membrane for BSA filtration (pre-hydrolysis treated)	FRR above ~95.2% (for the “M-3” membrane) over filtration cycles; RFD ~19.4–21.0%.	[34]
PAN membrane for oil/toluene solution (P(AN-co-MA))	TFR ~95%; RFR ~74%; IFR ~21%; FRR ~79%.	[35]
PAN vs. other polymers (PES, PVDF, PS) in digestate ultrafiltration	FRR of the PS, PVDF, PES, and PAN membranes were only 11.1%, 8.6%, 7.9%, and 6.2%	[36]
PAN/PU blend membranes (pure PAN and blends)	Pure PAN: FRR ~88%; 70/30 PAN/PU blend: FRR 99%	[37]
ACF (22.5%)	FRR: 82.3%, DRr 8.9% and DRir 3.7%	This work

## Data Availability

The original contributions presented in this study are included in the article. Further inquiries can be directed to the corresponding author.
